# Secondary Amputation After Failed Limb-Salvage Surgery Shows Comparable Long-Term Oncological Outcomes to Primary Amputation in Extremity Sarcoma: A 5-Year Follow-Up Study

**DOI:** 10.3390/jcm14124074

**Published:** 2025-06-09

**Authors:** Ortal Segal, Guy Ben Arie, Solomon Dadia, Ofer Marimsky, Assaf Albagli, Yair Gortzak, Amit Benady, Ben Efrima

**Affiliations:** 1 National Department of Orthopaedic Oncology, Tel Aviv Sourasky Medical Center, Tel Aviv 6423906, Israel; yairg@tlvmc.gov.il; 2 Levin Center for 3D Printing and Surgical Innovation, Tel Aviv Sourasky Medical Center, Tel Aviv 6423906, Israelamitbe@tlvmc.gov.il (A.B.); 3 Tel Aviv Sourasky Medical Center, Tel Aviv University, Tel Aviv 6423906, Israel; 4 Department of Foot and Ankle, Orthopaedic Surgery Division, Tel Aviv Sourasky Medical Center, Tel Aviv 6423906, Israel; assafal@tlvmc.gov.il (A.A.); bene@tlvmc.gov.il (B.E.)

**Keywords:** secondary amputation, limb salvage, sarcoma, primary amputation, post-amputation metastasis

## Abstract

**Aims:** Extremity sarcomas (ES) are rare, aggressive malignancies requiring complex surgical decisions. While limb-salvage surgery (LSS) is the preferred treatment to preserve functionality, local disease progression can necessitate secondary amputation. The oncological outcomes of secondary amputation compared to primary amputation remain uncertain, particularly with long-term follow-up. This study aimed to compare overall survival (OS), metastasis-free survival (MFS), local recurrence-free survival (LRFS), and postoperative complications between ES patients undergoing primary amputation (V1) and those requiring secondary amputation after failing LSS (V2), with a minimum follow-up of five years. **Methods:** A retrospective review was conducted at a national sarcoma center, including 71 patients treated between 2007 and 2017. Patients were categorized into V1 (n = 28) and V2 (n = 43) groups. Clinical and oncological data were collected from medical records and imaging, including tumor stage, surgical margins, and postoperative complications. All patients were followed up for a minimum of five years or until death. Kaplan–Meier survival analysis was performed to evaluate OS, MFS, and LRFS. **Results:** OS was 25% in the V1 group and 39.5% in the V2 group (*p* = 0.6). MFS (10.5 months, *p* = 0.2) and LRFS (27.4 vs. 34.4 months, *p* = 0.6) were comparable between groups. Postoperative complications occurred in 34.9% (V1) and 32.1% (V2) of patients, with infections being the most common complication. Patients with complications exhibited shorter MFS (*p* = 0.029). Negative surgical margins were achieved at 96.4% (V1) and 97.6% (V2). **Conclusions:** Secondary amputation following failing LSS demonstrates similar oncological outcomes to primary amputation, even with a minimum follow-up of five years. These findings support LSS as the preferred initial approach for ES patients. Postoperative complications associated with reduced MFS underscore the need for rigorous postoperative protocols. A multidisciplinary approach remains essential for optimizing long-term outcomes.

## 1. Introduction

Sarcomas are rare and aggressive malignant tumors, accounting for approximately 1% of all adult cancers. More than 50% of sarcomas occur in the extremities, with over 70% located in the lower limbs. The prognosis for extremity sarcomas (ES) depends on several factors, including patient age, preexisting medical conditions, tumor size and grade, histological subtype, lymph node involvement, and metastasis. Overall, sarcomas have a poor prognosis, with a five-year survival rate of around 50%. A key factor for optimizing overall survival (OS) is tailoring patient-specific surgical treatment that incorporates achieving negative surgical margins while maximizing limb functionality.

Limb amputation (AS) for ES was introduced in the 19th century, and, for many years, was the gold standard of treatment. However, it was associated with high mortality rates and poor functional outcomes. With the advent of multidisciplinary approaches, including neoadjuvant and adjuvant chemotherapy, radiotherapy, advanced imaging, and improved surgical techniques, the treatment paradigm has shifted toward limb-salvage surgery (LSS), which sometimes has its complications, such as local disease progression. Today, AS is generally reserved for cases where obtaining tumor-free margins with LSS is impossible, where limb functionality cannot be preserved, for palliative care, or for secondary amputation in LSS patients suffering from local disease progression.

The current consensus advocates LSS as the primary option. However, some studies argue that this trend may have shifted too far, and the possibility of secondary amputation after LSS and its risks should be factored into the decision-making process. With ongoing advancements in treatment, it is essential to reassess the surgical decision-making process periodically. Our study hypothesized that LSS resulting in local disease progression and subsequent secondary amputation yields different oncological outcomes compared to primary amputation. This single-center study, conducted at a national sarcoma center over the course of a decade, aims to compare OS, local recurrence-free survival (LRFS), metastasis-free survival (MFS), and complication rates between primary and secondary AS. Additionally, the study evaluates the impact of surgical margins and type of amputation on prognosis.

## 2. Materials and Methods

### 2.1. Patient Selection

This retrospective study was conducted between January 2007 and December 2017 at a national sarcoma center and was approved by the institutional review board. A total of 71 consecutive patients were included. The inclusion criteria were sarcoma patients over the age of 18 who were treated with either primary amputation surgery or secondary amputation as a result of local disease progression after LSS. Patients under 18 years of age and those who had undergone amputation for other types of tumors were excluded. All patients were evaluated by a multidisciplinary cancer board consisting of oncologists, radiologists, pathologists, and orthopedic surgeons specializing in surgical oncology. A high-volume orthopedic surgeon with expertise in surgical oncology performed all of the surgeries.

### 2.2. Preoperative Evaluation

Preoperative evaluation included pathological confirmation of the diagnosis, with sarcoma subtypes broadly categorized as either soft tissue or bone sarcomas, as determined by biopsy. Preoperative imaging, including CT or MRI of the affected limb, was conducted to assess tumor location, size, composition, and proximity to vital structures. Staging was performed using either total-body CT or PET-CT.

### 2.3. Postoperative Evaluation

Follow-up evaluations included clinical examinations and imaging, either total-body CT or PET-CT. During the first year, these were conducted at intervals of 3–6 months and then annually, continuing for five years or until the patient passed away.

### 2.4. Methods

Patient demographics were collected, and medical records were reviewed. Preoperative imaging was analyzed to assess tumor location and size. Tumors were categorized into four groups based on location: below the knee (BK), above the knee and below the pelvis (AK), hip and pelvis (PEL), and upper limb (UL). PET-CT or total-body CT was used to assess the presence and location of preoperative metastasis.

Tumor staging was performed using the American Joint Committee on Cancer (AJCC) Cancer Staging Manual, and tumors were classified into stages I–IV. The use of neoadjuvant chemotherapy and radiotherapy was also documented. Patients were divided into two surgical groups: primary amputation (V1) and secondary amputation (V2). The type of amputation was also recorded. Pathology reports were used to evaluate surgical margins, which were classified according to the R classification (R0, R1, and R2). Postoperative data were analyzed, including OS, MFS, LRFS, metastasis location, and complications. The primary outcome was to compare OS, MFS, LRFS, metastasis location, and complications between the V1 and V2 groups. Outcome data were collected by independent assessors who were blinded as to whether each amputation was primary or secondary.

## 3. Results

A total of 71 patients (28 in group V1 and 43 in group V2) were included in this study, with a mean follow-up time of 41 ± 42 months. Patient demographics and primary tumor locations are detailed in Table 1.

This table presents the baseline demographics and tumor characteristics of the study cohort, including age, gender, tumor type, and location. Data are stratified by primary amputation (V1) and secondary amputation (V2) groups.

Preoperative staging, presence of preoperative metastasis, and details regarding adjuvant and neoadjuvant treatments are reported in Table 2. No statistically significant differences were found in the two groups’ prevalence or location of metastasis.

Regarding amputation procedures, 9 patients underwent below-knee amputation (BKA) (17.8% in V1 and 16.3% in V2), while 23 patients had above-the-knee amputation (AKA) (35.7% in V1 and 30.2% in V2). Disarticulation was performed in 35 patients (42.9% in V1 and 53.5% in V2), and four patients had forequarter amputation (14.3% in V1 only).

Negative (R0) surgical margins were achieved in 96.4% of V1 patients and 97.6% of V2 patients. Two patients, one from each group, had positive surgical margins.

This table summarizes preoperative staging and treatment details, including tumor stage, presence of metastases, and use of neoadjuvant or adjuvant therapies. Comparisons between the primary amputation (V1) and secondary amputation (V2) groups are included.

Postoperative complications were observed in 24 patients (34.9% in the V1 group and 32.1% in the V2 group). The most common complication in both groups was postoperative infection, accounting for 56% of complications in V1 and 60% in V2. A detailed breakdown of postoperative complications is provided in Table 3. The mean time to complication onset was 6.9 ± 12.6 months, with a group-specific mean of 9.5 ± 16 months in V1 and 5.2 ± 10 months in V2. Local recurrence was observed in four patients (7% in V1 and 3.6% in V2). Among patients with initial metastasis-free staging, 23 developed postoperative metastases (36% in V1 and 30% in V2), with the lung being the most common metastatic site. Table 3 also provides details on the locations of postoperative metastasis.

The overall survival (OS) rate was 33.8% (25% in V1 and 39.5% in V2), with a mean time to death of 23.4 ± 23.3 months (22.5 ± 18.6 months in V1 and 23.9 ± 27 months in V2). No statistically significant differences were observed between primary and secondary amputation in terms of metastasis-free survival (MFS), OS, time to death, complication rates, or local recurrence-free survival (LRFS). The type of amputation, whether primary or secondary, did not influence the time to recurrence or time to death. The preoperative staging did not affect the time to death or time to metastasis, and postoperative complications were not associated with differences in time to death. These comparative postoperative outcomes are summarized in Table 3.

## 4. Discussion

Advances in surgical techniques, neoadjuvant and adjuvant therapies, improved imaging modalities, and implant technology have significantly shifted the treatment paradigm for ES, moving from radical limb amputations to LSS. Research consistently shows that LSS leads to superior functionality and an enhanced quality of life for patients. However, approximately 14% of patients who undergo LSS eventually require secondary amputation. This raises the question of whether secondary amputation results in a worse prognosis compared to primary amputation [1,2,3,4,5]. The key finding of this study is that there were no significant differences in complication rates, incidence of new metastases, or local recurrence between the two groups. Furthermore, OS, LRFS, and MFS did not differ significantly, which is consistent with existing literature [6,7,8,9,10]. Notably, preoperative staging did not impact survival outcomes, whereas patients who experienced postoperative complications had significantly shorter MFS.

While LSS is increasingly favored for treating ES, there is conflicting evidence regarding its superiority over AS in terms of OS in specific clinical settings. He et al.’s meta-analysis demonstrated that patients who underwent LSS had significantly better OS compared to those treated with AS [7]. Similarly, Abdelgawad et al. found that LSS was associated with notably higher OS than AS. However, Han et al.’s meta-analysis reported a favorable odds ratio for AS, suggesting that AS may offer better survival in certain cases [8]. In our cohort, the OS rate for amputations was 33.8%, with the V1 group at 25% and the V2 group at 39.5% (*p* = 0.6) showing no significant survival advantage for AS. These findings reflect the ongoing debate but support the value of LSS in improving long-term survival while preserving functionality in ES patients, even with the risk of local disease progression and secondary amputation.

Postoperative metastasis and local disease progression are critical considerations when determining the optimal surgical approach for ES. The choice between LSS and AS hinges on balancing oncological control with functional outcomes. According to Kirilova et al., 30% of patients who underwent AS for ES developed metastases [4]. In contrast, Kaneuchi et al. reported metastasis rates of 28% in LSS patients and 36% in AS patients, while Baysal et al. found similar metastasis rates in AS patients [11,12]. In our study, metastasis occurred postoperatively in 36% of patients in the V1 group and 30% in the V2 group, with no statistically significant difference between the two groups. The mean MFS was 10.5 months. This aligns with previous studies, which indicated that the risk of metastasis is comparable between LSS and AS [13,14,15]. Additionally, it underscores that within the first-year post-surgery, the rates of metastasis are comparable for both primary and secondary amputations.

The emergence of lung metastases following amputation may be influenced by factors such as immunosuppression from surgical stress and the effects of wound-healing cytokines. Given that 93% of our cohort presented with advanced disease (stage III or above), a possible explanation could be the presence of preoperative micrometastases [16,17,18,19]. The importance of detecting micrometastases has been discussed extensively in the literature. Some authors have suggested that circulating tumor DNA (ctDNA) could help identify micrometastases more effectively, potentially guiding neoadjuvant and adjuvant treatment strategies. However, the specific role of ctDNA testing in the management of ES still needs to be determined [17].

Local disease progression after LSS has been reported to range from 15% to 20% in the literature [20,21,22,23,24,25,26]. Abdelgawad et al. highlighted significantly higher local disease progression rates in LSS compared to AS, reinforcing concerns about the oncological outcomes associated with limb-salvage techniques. In contrast, in our study, no statistically significant difference in local recurrence between V1 and V2 was found (*p* = 0.6). Given the absence of significant differences in both metastasis and local recurrence between V1 and V2, surgical decision-making should prioritize functional preservation and the achievement of clear surgical margins. Our finding demonstrated that only one patient in each group had positive surgical margins, indicating that free surgical margins are obtainable in both V1 and V2 groups; therefore, LSS should be attempted if possible.

The difference in MFS between primary and secondary amputation patient groups is not extensively detailed in the literature. However, several studies provide relevant insights into oncological outcomes and the associated survival rates of these two surgical approaches [27,28]. Kirilova et al. and Erstad et al. reported no statistically significant differences in metastatic disease between patients undergoing primary amputation and those receiving secondary amputation [4,28]. Similarly, our study’s mean difference in MFS between the V1 and V2 groups was not statistically significant (*p* = 0.23), highlighting that disease advancement after LSS resulting in secondary amputation will not compromise the oncological outcomes. This suggests that LSS remains a preferable option due to its preservation of functional status without compromising survival.

Notably, patients who experienced postoperative complications exhibited shorter MFS (*p* = 0.029). This could be attributed to factors such as a pro-inflammatory state, the survival of residual tumor cells, poor overall health, and delays in initiating adjuvant therapy. Importantly, no significant difference in postoperative complications was observed between the V1 and V2 groups, underscoring that LSS can be attempted, even with the risk of secondary amputation due to local disease progression, without reducing MFS. For surgeons in both groups of patients, a higher level of vigilance is warranted when postoperative complications arise, as these may correlate with early metastasis. In such cases, early imaging might be beneficial. Furthermore, patients should be informed that the presence of postoperative complications may be associated with a poorer overall prognosis.

Our finding that postoperative complications, mainly infections, were associated with shorter MFS contrasts with the results of Jeys et al., who reported improved survival in patients with wound infections after surgery for high-grade localized osteosarcoma [29]. They attributed this to a possible anti-tumor immune response triggered by infection. In contrast, our cohort included heterogeneous sarcoma subtypes, predominantly with advanced-stage disease, where immunosuppression, delays in adjuvant therapy, or systemic inflammation may have contributed to earlier metastasis. These divergent findings underscore the need for further research to clarify the immunologic and therapeutic dynamics following postoperative complications in sarcoma patients.

This study has several limitations that should be considered when interpreting the results. First, as a retrospective single-center study, it is subject to inherent biases, such as selection bias and reliance on incomplete or inconsistently recorded medical data, which may affect the accuracy of the findings. Additionally, the relatively small sample size of 71 patients may limit the statistical power to detect smaller but clinically meaningful differences between primary and secondary amputation groups. Patients undergoing secondary amputation may represent a more complex and advanced disease cohort, which could influence the outcomes. Furthermore, the mean follow-up period may be insufficient to capture long-term outcomes such as OS, MFS, and LRFS. Another limitation is the lack of functional and quality-of-life assessments, which are important considerations in the decision between LSS with a risk of secondary amputation and primary amputation. The inclusion of multiple sarcoma subtypes adds heterogeneity to the sample, potentially obscuring subtype-specific outcomes. Finally, because the study was conducted in a single high-volume national sarcoma center, the findings may not be generalizable to other healthcare settings with different resources or expertise. These limitations suggest the need for further multicenter studies with larger sample sizes and longer follow-up periods to validate these findings and explore functional outcomes.

In conclusion, secondary amputation following failing limb-salvage surgery (LSS) demonstrated comparable overall survival (OS), metastasis-free survival (MFS), and local recurrence-free survival (LRFS) to primary amputation in patients with extremity sarcoma (ES). These results indicate that secondary amputation, performed after LSS and subsequent local disease progression, does not result in worse outcomes compared to primary amputation. This supports prior studies advocating for LSS over primary amputation as the initial approach. Additionally, postoperative complications were associated with shorter MFS, highlighting the need for more rigorous postoperative staging protocols for patients experiencing immediate postoperative complications.

## 5. Clinical Relevance

1. Postoperative complications: postoperative complications were associated with reduced MFS, underscoring the need for careful monitoring.
2. Secondary amputation: outcomes for secondary amputation after failing LSS are comparable to primary amputation, affirming LSS as a strong initial treatment.

## Figures and Tables

**Table 1:** Patient demographics and tumor characteristics.

Category	V1	V2	Total	*p*-Value
Total Patients	28	43	71	-
Gender	20 M/8 F	32 M/11 F	52 M/19 F	0.8
Mean Age (Years)	47 ± 26	54 ± 24	51 ± 25	0.3
Primary Tumor Location—Below the Knee	9 (32.10%)	14 (32.60%)	23 (32.4%)	-
Primary Tumor Location—Above the Knee	9 (32.10%)	21 (48.80%)	30 (42.2%)	-
Primary Tumor Location—Pelvis	6 (21.90%)	8 (18.60%)	14 (19.7%)	-
Primary Tumor Location—Upper Limb	4 (14.30%)	0 (0%)	4 (5.6%)	-
Tumor Type—Bone Sarcoma	16 (57.1%)	20 (46.5%)	36 (50.7%)	-
Tumor Type— Soft Tissue Sarcoma	12 (42.9%)	23 (53.5%)	35 (49.3%)	-

**Table 2:** Preoperative staging and treatment details.

Category	V1	V2	Total	*p*-Value
Stage I or II	3 (10.70%)	1 (2.30%)	4 (5.6%)	0.4
Stage III	15 (53.60%)	26 (60.50%)	41 (56.3%)	0.4
Stage IV	10 (35.70%)	16 (37.20%)	26 (36.6%)	0.4
Preoperative Metastasis—Total	10 (35.7%)	14 (32.5%)	24 (33.8%)	0.8
Preoperative Metastasis—Lungs	10 (35.7%)	10 (23.2%)	20 (28.1%)	0.2
Preoperative Metastasis—Other: Abdomen or Bone	1 (1.4%)	5 (11.6%)	6 (8.4%)	0.2
Neoadjuvant/Adjuvant Chemotherapy	20 (71.4%)	29 (67.4%)	49 (69.0%)	0.8
Neoadjuvant/Adjuvant Radiotherapy	8 (28.5%)	20 (46.5%)	28 (39.4%)	0.1

**Table 3:** Surgical outcomes and postoperative complications.

Category	V1	V2	Total/Notes	*p*-Value
Postoperative Complications	34.90%	32.10%	24 patients (most common—infection)	0.6
Time to Complication (Months)	9.5 ± 16	5.2 ± 10	Mean: 6.9 ± 12.6	0.5
Postoperative Metastasis	36%	30%	Most common location—lungs (32.4% overall)	0.8
MFS (Months)	10 ± 7.6	10.8 ± 15.8	Mean: 10.5 ± 12.7	0.2
Local Recurrence (LR)	7%	3.60%	4 (5.6%)	-
LRFS (Months)	27.4 ± 26.6	34.4 ± 36.1	Mean: 31.7 ± 32.6	0.6
Overall Survival (OS)	25%	39.50%	33.80%	0.3
Time to Death (Months)	22.5 ± 18.6	23.9 ± 27	Mean: 23.4 ± 23.3	0.6

This table outlines the surgical outcomes and postoperative complications, including rates of infection, local recurrence, and metastasis. It also details the time to complication onset and compares outcomes between the primary (V1) and secondary (V2) amputation groups. MFS—Metastasis Free Survival, LRFS—Local Recurrence Free Survival.

## Data Availability

The original contributions presented in this study are included in the article. Further inquiries can be directed to the corresponding author.
